# Repetitive transcranial magnetic stimulation treatment for peripartum depression: systematic review & meta-analysis

**DOI:** 10.1186/s12884-021-03600-3

**Published:** 2021-02-09

**Authors:** Hyune June Lee, Sung Min Kim, Ji Yean Kwon

**Affiliations:** Department of Medical Devices Industry, Dongguk University, Seoul, South Korea

**Keywords:** Repetitive transcranial magnetic stimulation, rTMS, Peripartum depression, Pregnancy

## Abstract

**Background:**

Peripartum depression is a common disorder with very high potential hazards for both the patients and their babies. The typical treatment options include antidepressants and electroconvulsive therapy. However, these treatments do not ensure the safety of the fetus. Recently, repetitive transcranial magnetic stimulation has emerged as a promising treatment for neuropathies as well as depression. Nevertheless, many studies excluded pregnant women. This systematic review was conducted to confirm whether repetitive transcranial magnetic stimulation was a suitable treatment option for peripartum depression.

**Methods:**

We performed a systematic review that followed the PRISMA guidelines. We searched for studies in the MEDLINE, PsycINFO, EMBASE, and Cochrane library databases published until the end of September 2020. Eleven studies were selected for the systematic review, and five studies were selected for quantitative synthesis. Data analysis was conducted using Comprehensive Meta-Analysis 3 software. The effect size was analyzed using the standardized mean difference, and the 95% confidence interval (CI) was determined by the generic inverse variance estimation method.

**Results:**

The therapeutic effect size of repetitive transcranial magnetic stimulation for peripartum depression was 1.394 (95% CI: 0.944–1.843), and the sensitivity analysis effect size was 1.074 (95% CI: 0.689–1.459), indicating a significant effect. The side effect size of repetitive transcranial magnetic stimulation for peripartum depression was 0.346 (95% CI: 0.214–0.506), a meaningful result. There were no severe side effects to the mothers or fetuses.

**Conclusions:**

From various perspectives, repetitive transcranial magnetic stimulation can be considered an alternative treatment to treat peripartum depression to avoid exposure of fetuses to drugs and the severe side effects of electroconvulsive therapy. Further research is required to increase confidence in the results.

**Supplementary Information:**

The online version contains supplementary material available at 10.1186/s12884-021-03600-3.

## Background

Peripartum depression (PPD) is defined as major depression occurring within the gestational period and 4 weeks after childbirth [1]. PPD is a common disease that 10 ~ 15% of pregnant women experience (https://www.who.int/mental_health/maternal-child/maternal_mental_health/en/), and PPD patients incur a massive lifetime expense of 75,728 pounds (USD $95,656) [2]. PPD threatens not only the mother’s health by causes hormonal imbalance [3], alcohol and substance abuse [4], and more than 5-fold increased maternal suicide [5]. The health of the fetus is also affected by premature birth (the effect size of peripartum depression on premature birth is 1.38) [6], and low birth weight (the effect size of peripartum depression on low birth weight is 1.3) [7]. PPD also interferes with creating a stable attachment in infants ‘growth process, which can result in self-control and cognitive function behavioral issues [8].

The methods for treating PPD are mainly electroconvulsive therapy (ECT) and antidepressants [9]. Although both have proven treatment effectiveness for PPD [10–13], some concerns for the safety of pregnant women and fetuses have been raised. Antidepressants relieve depression by regulating the neurotransmitters that make people feel happy [14]. Neurotransmitters released from pre-synaptic neurons are reabsorbed or decomposed by monoamine oxidase enzymes to transfer them to the neurons from the synapses Antidepressants reduce neurotransmitter decomposition by interfering with their reabsorption and the activity of monoamine oxidase enzymes, thereby alleviating depression [15]. Antidepressants are one of the most used treatment methods for PPD, but there are some concerns. Because components of antidepressants can pass through the placenta [16], these could have a chemical effect on the fetus. Pregnant women taking SSRI have a 7-fold increased risk of spontaneously induced abortion [17, 18], more than 3-fold increased risk of fetal infections [19], and increased risks of premature birth and underweight babies [20], autism spectrum disorder in the baby [21], increased risk of motor, speech, and scholastic disorder [22], cardiac defects [23] and persistent pulmonary hypertension [24]. Also, infants can be exposed to antidepressants and their metabolites during breastfeeding [25], which could increase monoamine oxidase levels and affect the functional maturity of the infant’s brain [26]. Side effects, such as decreased feeding [27], colic, and irritability [28] have been reported. Another treatment, ECT, is delivering a stimulating electrical shock to the brain to relieve depression. Electric shocks affecting neurons and chemicals in the brain produce short and controlled seizures, which have excellent effects on various neurological disorders [29]. However, treating pregnant women with ECTs can cause adverse effects such as vaginal bleeding and miscarriage [30], uterine contractions [31], abdominal pain [32], and preeclampsia [33]. Clinicians recommend these treatments because the risk of the treatment is smaller than the risk of PPD [34, 35] and their effectiveness has been proven. However, considering these treatment’s safety concerns for the fetus and mother, research to identify safer treatment methods is needed.

With the recent development in brain stimulation research, many researchers are increasingly trying to use brain stimulation for various neurological treatments [36]. One type of brain stimulation, repetitive transcranial magnetic stimulation (rTMS), stimulates the brain’s dorsolateral prefrontal cortex (DLPFC) with magnetic fields to induce the degeneration of neurons and activate the neural system of the brain to relieve depression [37]. Using rTMS to treat PPD is still reluctant [38–40], although studies have shown that rTMS is safe [41] and effective for treating several neurological diseases [42–44].

Thus, in this paper, we conducted a systematic review and meta-analysis to confirm whether rTMS treatment was suitable for PPD. There are few systematic review studies on rTMS treatment for PPD [45–48], but no studies statistically analyzed the therapeutic effects and safety and considered the adverse effects on fetuses. To investigate these problems, we conducted a meta-analysis to confirm the effect size of the therapeutic effects and safety and extended the range of patients from pregnant patients to 1 year after childbirth to identify the effect on the fetuses.

## Methods

The systematic review followed the PRISMA guidelines (registration number CRD42020197855) [49].

### Key questions

The criteria used in selecting the relevant studies included patients from the pregnancy period to 1 year after childbirth, intervention with TMS, no limits on the comparators, and outcomes measured by the degree of depression alleviation. The detailed key questions were:

(1) Is rTMS effective for PPD? What is the effect size of rTMS for PPD?

(2) Does rTMS have maternal and fetal side effects? What is the effect size of the side effects caused by rTMS?

### Inclusion and exclusion criteria

In general, the studies used for the meta-analysis were randomized controlled trials (RCTs), but non-randomized studies (NRS) were also included in the meta-analysis, and case studies were included in the systematic review because only a small number of randomized trials provided evidence of the effects of the interventions [50] associated with pregnancy. Before inclusion, the non-randomized studies were evaluated to verify that the patients, interventions, comparisons, and outcomes were set appropriately by the Cochrane algorithm. If systematic reviews or meta-analyses were included, we checked the included studies and references. The criteria for exclusion were (1) experimental studies with animals, (2) studies not published in English, (3) studies that were not original (if there was only abstract, the original text was requested in an e-mail to the author), (4) symptoms of baby blues or postpartum psychosis, and (5) major depressive disorder that did not occur from pregnancy to 1 year after childbirth.

### Literature search strategy

**Fig. 1:**
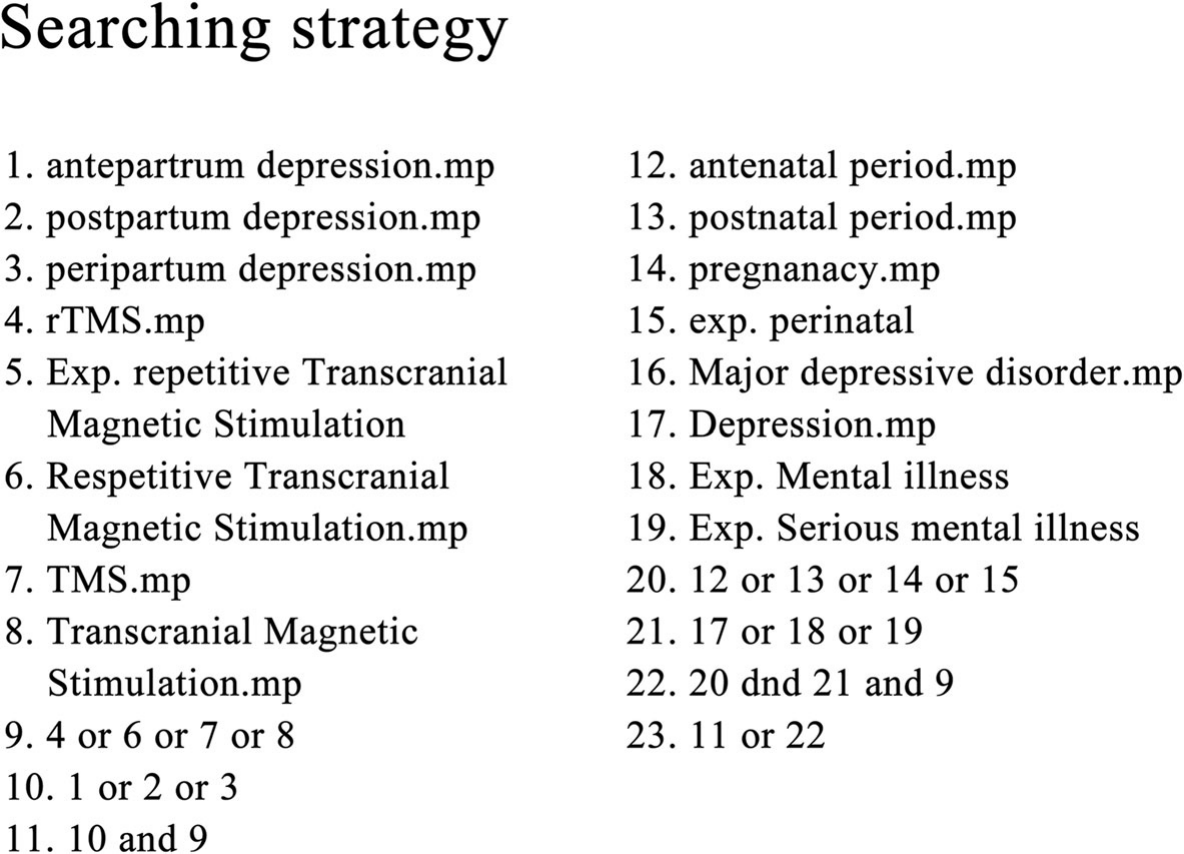
Searching strategy

We performed a literature search of studies published before the end of September 2020 using EMBASE, MEDLINE, PsycINFO, and the Cochrane Library. In the literature search, the medical indications and treatment methods were searched using Boolean models. The indications search terms were using peripartum depression or antepartum depression or postpartum depression or pregnancy or perinatal, and the treatment searches were using rTMS or repeated transcranial magnetic stimulation or TMS or transcranial magnetic stimulation. If there were mapping or mesh term, it was used, otherwise it was extensive mode. The detailed search strategies are presented in Fig. 1. To minimize the omission of data and increase reliability, HJL, and JYK independently reviewed the literature and, if the opinions of the researchers differed, the studies were reviewed together, and an agreement was reached.

### Data extraction

For the final selected literature, the following data were extracted: basic information (the title of paper, author, year of publication), study characteristics (study design, number of subjects), subject information (age, primary psychiatric diagnosis, gestational age, and simultaneous treatment), treatment parameters (motor threshold, site of stimulation, frequency and pulse, interval time, number of sessions) [51] and the result (outcome measure at baseline and at the end of treatment, side effects on mothers and fetuses).

### Risk of bias assessment

After the literature screening, a bias evaluation of the studies was performed by two evaluators independently to clarify ambiguous differences between the reporting quality and research quality. Different evaluation methods were applied according to the study design. RCT studies were examined using the risk-of-bias 2 (ROB2) tool [52], and the NRS were examined using the Risk of Bias Assessment Tool for Non-randomized Studies (ROBINS-I) [53]. Case reports and series were evaluated used the methodological quality tool [54] because they could be adopted as evidence of new treatments [55]. The final judgment on the overall risk of bias was agreed upon between the two evaluators, and publication bias was visually reviewed through funnel plots, and additionally, statistically confirmed through Egger’s regression.

### Data analysis

Data analysis was conducted using Comprehensive Meta-Analysis 3 software (CMA3). The effect size was used standardized mean difference (SMD) was obtained using two-group, pre-post data in the RCTs, and one group pre-post data for the NRS [56]. For the 95% CI and the correlation coefficient value of 0.5, the generic inverse variance estimation method was used [57]. Safety was assessed using the effect size of the side effects, and the effect size was calculated by the same method as the treatment effect. The calculations are presented as a Forest plot. Heterogeneity was determined using the statistical test method Cochrane’s Q test and the I-squared (I^2^) statistic. If the *p*-value of the Q test value exceeded 0.1 and the I^2^ statistic exceeded 50%, a random-effects model was applied, and if the Q test value was less than 0.1 and the I^2^ statistic was less than 50%, a fixed-effects model was applied.

## Results

### Search results

**Fig. 2:**
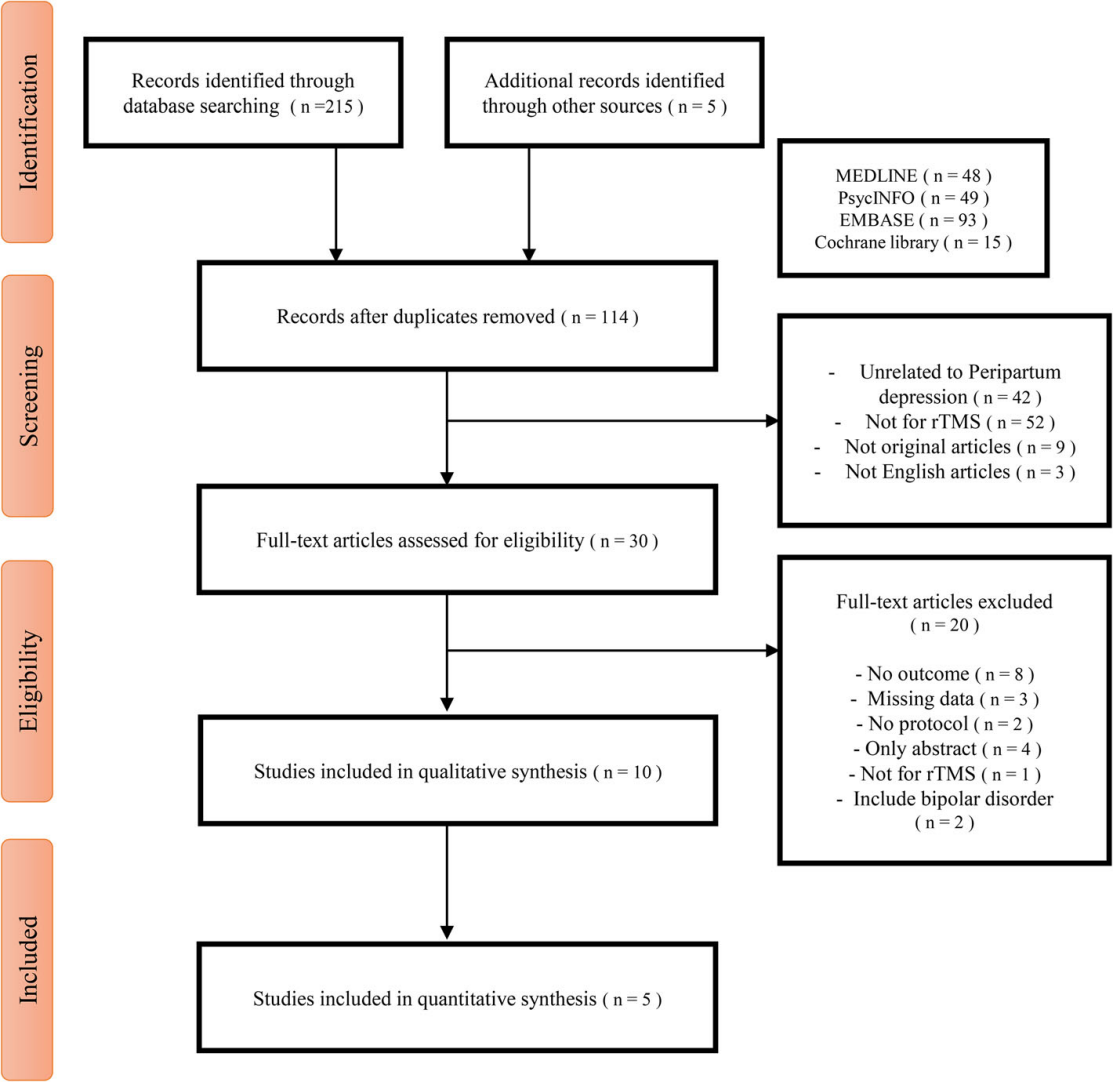
Study Selection PRISMA flow diagram

We conducted a literature search following the PRISMA FLOW guidelines (Fig. 2). Of the 229 studies identified in the database, 128 remained after the removal of the duplicates. Ninety-eight studies were excluded because they did not meet the inclusion and exclusion criteria. We performed a full-text review of 30 studies to confirm whether they were suitable for our research purposes. Finally, 10 studies suitable for the systematic review, and five studies suitable for meta-analysis were selected to confirm the therapeutic effect and safety of rTMS. The detailed of exclude studies and reason is presented Additional files 1. There were three studies with only abstracts, so we requested the original text by email, but no author responded.

### Characteristics of the selected studies

Eleven studies identified the efficacy and safety of rTMS for PPD, two RCT studies [58, 59], four NRS [60–62], and five case studies [63–67]. One NRS study was excluded from the meta-analysis since the study did not report detailed outcomes. The total number of participants was 101, 84 of whom received active rTMS treatment. During pregnancy, 65 patients were treated with rTMS, and most of them were in the second and third trimesters of pregnancy. Seventeen patients were treated for postpartum depression, and two patients were treated during pregnancy and after delivery. Although 20 participants were treated with antidepressants [60, 61, 65] along with rTMS, their antidepressant doses were stable for two to 4 weeks, and one participant was treated with clonazepam for insomnia [62]. The treatment parameters and protocols in each study were all different, which seemed to be because the protocol for TMS therapy for perinatal depression has not yet been accurately established. Twenty-two patients were stimulated to the right DLPFC [58, 60, 65, 66], 79 to the left DLPFC [59, 61–65], and the patients in one studies [65] were stimulated on both sites. The patients who were stimulated on the right DLPFC showed symptoms of anxiety and were treated with a frequency of less than 1 Hz, which is relatively less than that used to stimulate the left DLPFC (more than 5 Hz). The researchers considered the interval time and duration of stimulation to minimize side effects such as seizures and did not show any common characteristics for the other treatment parameters. The detailed are presented Tables 1, 2, and 3.

**Table 1:** Characteristics of the subjects in the included studies

Study	Study design	Subjects	Age	Gestational age	Psychiatric diagnosis	Simultaneous treatment
D. R. Kim et al., 2019 [58]	Randomized controlled trial	Active 11	30.13 ± 5.78	22.19 ± 7.11 (Weeks)	MDD	Free
		Sham 11	26.41 ± 5.11	25.62 ± 7.61 (Weeks)		
Myczkowski et al., 2012 [59]	Double-blind randomized controlled trial	Active 8	29.63 ± 6.37	4.13 ± 2.85 (Month)	MDD	Free
		Sham 6	26.67 ± 7.15	3.50 ± 2.74 (Month)		
D. R. Kim et al., 2011 [60]	Non-randomized controlled trial	10	31.2 ± 5.6	25.8 ± 5.16 (weeks)	MDD	4 patients treated with antidepressants
Hizli Sayar et al., 2014 [61]	Non-randomized controlled trial	30	32.69 ± 3.69	14.26 ± 8.25 (weeks)	MDD	12 patients treated with antidepressants
Garcia et al., 2010 [62]	Non-randomized controlled trial	7	34.11 ± 6.05	After birth 30 days to 1 year	MDD	Free
Zhang et al., 2010 [63]	Case report	1	28	14 (weeks)	MDD	Free
Tan et al., 2008 [64]	Case report	1	30	From 0 to postpartum period	MDD	Free
Ferra˜o et al., 2018 [65]	Case report	3 (Left)	35.7 ± 2.05	6.67 ± 3.06	MDD	2 patients treated with antidepressants
		1 (Right)	36	8		1 patient treated with antidepressants
Cohen et al., 2008 [66]	Case report	1	30	Until 20 (weeks)	MDD	Free
Monika Klírová et al. [67]	Case report	1 (Left)	30	16	MDD	Treated with antidepressant
		1 (Right)	30	31	MDD	Treated with antidepressant

*Abbreviations*: *MDD* Major depressive disorder

**Table 2:** Characteristics of the treatments in the included studies

Study	Motor threshold	Site of stimulation	Frequency	Number of pulses	Inter-event interval	Number of Sessions
D. R. Kim et al., 2019 [58]	100%	Right DLPFC	1-Hz	900	60 s on 60 s off	20
Myczkowski et al., 2012 [59]	120%	Left DLPFC	5-Hz	1250	10 s on 20 s off	25
D. R. Kim et al., 2011 [60]	100%	Right DLPFC	1-Hz	300	60 s on 60 s off	20
Hizli Sayar et al., 2014 [61]	100%	Left DLPFC	25-Hz	1000	2 s on 30 s off	18
Garcia et al., 2010 [62]	120%	Left DLPFC	10-Hz	150	4 s on 26 s off	20
Zhang et al., 2010 [63]	90%	Left DLPFC	1-Hz	1200	20 s off	42
Tan et al., 2008 [64]	110%	Left DLPFC	25-Hz	1000	2 s on 28 s off	77
Ferra˜o et al., 2018 [65]	120%	Left DLPFC	10-Hz	3000	*	42.67
		Right DLPRC	1-Hz	1800		20
Cohen et al., 2008 [66]	110%	Right DLPFC	1-Hz	1600	*	1
Monika Klírová et al. [67]	100%	Left DLPFC	20-Hz	2000	2 s on 30 s off	15
		Right DLPRC	1-Hz	300	60 s on 60 s off	15

*Abbreviations*: *DLPFC* Dorsolateral prefrontal cortex; *s* Seconds

**Table 3:** Characteristics of the outcomes in the included studies

Study	Instrument	Pre-TMS****	Post-TMS	Remission	Response	Side effects (mother)	Infants
D. R. Kim et al., 2019 [58]	HDRS-17	23.18 ± 3.54	9.27 ± 6.05	3	9	1 (10%)	4 (36%)
		22.27 ± 2.65	13.18 ± 8.00	2	5		
Myczkowski et al., 2012 [59]	HDRS-17	29.13 ± 5.64	18.50 ± 9.83	*	*	2 (25%)	0 (0%)
		26.67 ± 5.68	24.83 ± 7.60				
D. R. Kim et al., 2011 [60]	HDRS-17	24.4 ± 5.6	9.7 ± 6.1	3	7	4 (40%)	0 (0%)
Hizli Sayar et al., 2014 [61]	HDRS-17	26.67 ± 5.58	13.03 ± 6.93	6	12	0 (0%)	0 (0%)
Garcia et al., 2010 [62]	HDRS-24	22.67 ± 6.44	2.14 ± 3.19	8	9	2 (29%)	0 (0%)
Zhang et al., 2010 [63]	HDRS-24	35	8	1	1	0 (0%)	0 (0%)
Tan et al., 2008 [64]	HDRS-17	38	4	1	1	0 (0%)	0 (0%)
Ferra˜o et al., 2018 [65]	HDRS-21	24.33 ± 5.24	7.33 ± 4.03	2	3	2 (66%)	1 (25%)
		12	6	1	1	1 (100%)	
Cohen et al., 2008 [66]	HDRS-17	18	6	1	1	0 (0%)	0 (0%)
Monika Klírová et al., 2008 [67]	MADRS	33	2	*	*	0 (0%)	0 (0%)
	BDI	29	12	*	*		

*Abbreviations***:** *HDRS* Hamilton Depression Rating Scale (HDRS-21,24 is an expanded version of the HDRS-17), *MARDS* Montgomery-Åsberg Depression Rating Scale, *BDI* Beck Depression Inventory, *TMS* transcranial magnetic stimulation, *:  No information in paper

### Therapeutic effects

In the 10 studies selected for the systematic literature review, 76 patients had an average 59% improvement in depression. Thirty-seven percent of the participants showed depression remission (depression scale rating was lowered to that of a normal person), and 66% of the participants showed a response to rTMS (depression scale score reduced more than 50% compared to before treatment). In Kim’s study [58], one of the RCT studies, 45.45% of the control group responded, whereas 81% of the experimental group responded, and the remission rate also showed a significant difference of 9% or more between the experimental group and the control group. Another RCT study also showed a difference in the depression scale of about 23% between the control group and the experimental group, showing a similar treatment effect to that of antidepressants [59]. In the NRS, the response rate of 56 participants was 33%, and the remission rate was 59%. In the study by Garcia et al. [62], the depression scale scores showed that all participants achieved remission, and all except one participant responded to rTMS. The other NRS also showed treatment effects, and in the case study, all nine participants responded to rTMS. All studies showed treatment effectiveness, but treatment effectiveness declined as the study design was refined.

**Fig. 3:**
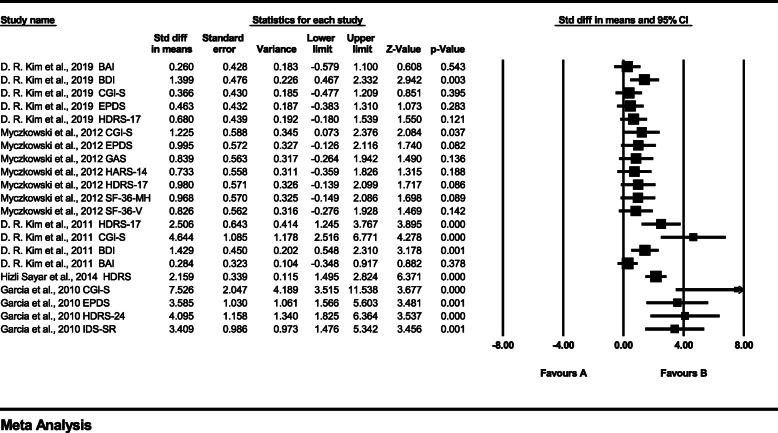
Forest plot of therapeutic effects. Abbreviations**:** BAI: Beck Anxiety Inventory; BDI: Beck Depression Inventory; CGI-S: Clinical Global. Impression Scale; EPDS: Edinburgh Postnatal Depression Scale; HDRS: Hamilton Depression. Rating Scale; GAS, Global Assessment Scale; SF-36-V and SF-36-MH: 36-item Quality of Life. Health Survey, Vitality, and Mental Health scores; IDS-SR: Inventory of Depressive. Symptomatology-Self-Report

Evaluation of the heterogeneity of the studies for treatment effects showed a *p-*value of < 0.001 and an *I* ^2^ statistic of 71.933. Because the *I* ^*2*^ statistic exceeded 50%, a random-effects model was applied (Fig. 3). The effect size of rTMS for PPD had an SMD of 1.394 (95% CI: 0.944–1.843), indicating a significant treatment effect (Z = 6.079, *p* < 0.01).

**Fig. 4:**
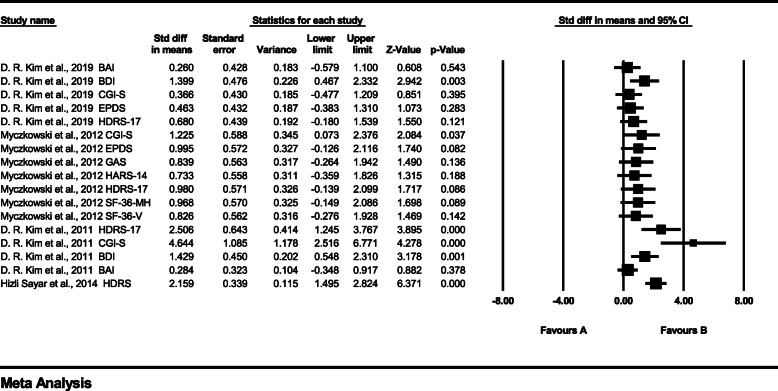
Forest plot of the sensitivity analysis Abbreviations: BAI: Beck Anxiety Inventory; BDI: Beck Depression Inventory; CGI-S: Clinical Global. Impression Scale; EPDS: Edinburgh Postnatal Depression Scale; HDRS: Hamilton Depression. Rating Scale; GAS, Global Assessment Scale; SF-36-V and SF-36-MH: 36-item Quality of Life. Health Survey, Vitality, and Mental Health scores

Figure 4 shows the sensitivity analysis performed because we thought that the treatment effect in one study [62] was measured too high. Excluding this, the heterogeneity showed a *p-*value of *< 0.01* and an *I*^*2*^ statistic of 62.593, and because the *I*^*2*^ value exceeded 50%, a random-effects model was applied. The effect size of rTMS for PPD had an SMD of 1.074 (95% CI: 0.689–1.459), indicating a meaningful treatment effect *(*Z = 5.468*, p* < 0.01*)*. The sensitivity analysis showed that the heterogeneity decreased, but the *I*^*2*^ value still exceeded 50%, and the treatment effect was also slightly reduced, but the treatment effect was still significant.

### Safety

In the 10 studies selected for systematic review, about 15% of the patients experienced side effects in the group receiving rTMS therapy. However, in the RCTs, there was no difference between the experimental groups and the control groups [58, 59], and there were minor side effects that did not worsen the patients’ health, such as headache [58, 60, 62, 65, 66] and pain at the stimulation site [62, 66]. Supine hypotension syndrome was reported as an adverse event in some mothers [60]. However, this is a disorder caused by posture during treatment and can be prevented after posture correction [68]. The side effects in the infants included preterm birth, and five fetuses experienced preterm birth, but all of the fetuses were healthy. Brachial plexus injury was reported as an adverse event in one fetus, but it was caused by the large shoulder of the fetus and was not a side effect of the treatment process [58].

**Fig. 5:**
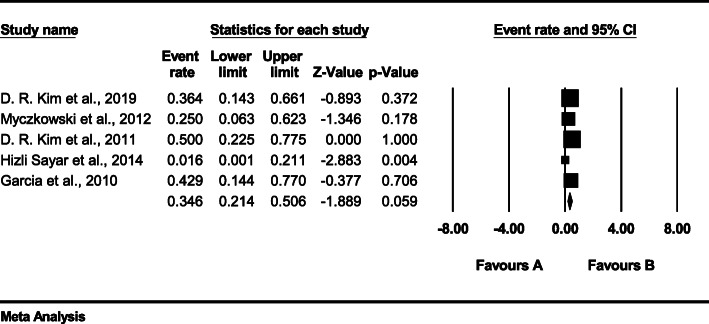
Forest plot of the side effects

Evaluation of the heterogeneity of the studies for the side effects showed a *p*-value of < 0.01 and an *I*^*2*^ value of 46.631 and, because the *I*^*2*^ value was less than 50%, a fixed-effects model was applied (Fig. 5) The effect size of side effects had an SMD of 0.346, (95% CI*:* 0.214–0.506), indicating that rTMS had an effect on the occurrence of side effects (Z = − 1.889, *p* = 0.059).

### Risk of bias

**Fig. 6:**
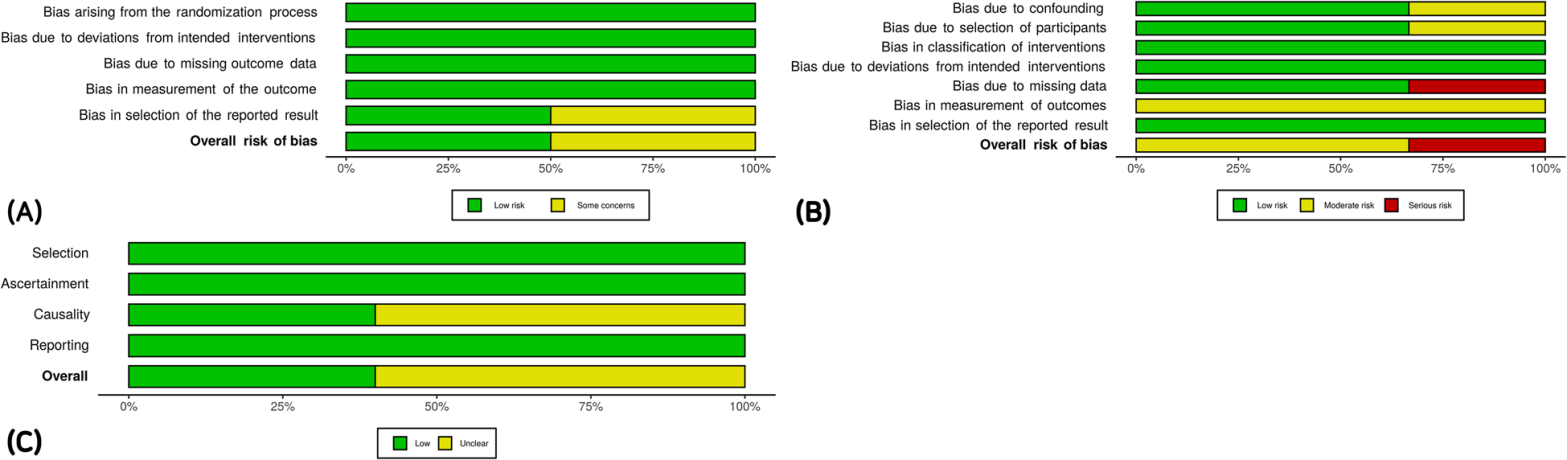
Risk of bias graphs: **a** RCT risk of bias, **b** NRS risk of bias, and **c** case study risk of bias

According to the experimental design, the RCTs were evaluated by ROB2, the NRS by ROBIN-I, and the case studies were evaluated using a methodological quality tool. We evaluated the bias according to the guidance of the tool and assessed the overall bias as a low risk of bias if all domains of bias were low risk, a high risk of bias if there were one or more high-risk domains or many suspected risks (Fig. 6). In the case of Myczkowski’s study [58], the study could have selective reporting bias because the planned outcomes were selectively reported. Bias in the outcomes measurements in the NRS was evaluated as moderate [60–62] because the study design of all of the NRS was open-label, meaning that the patients knew their treatment, and the risk of bias in Garcia’s study [62] was evaluated as high because there were missing data, which could have a significant impact on the outcome. The overall evaluation of bias was moderate, but this must be considered as it can cause over or underestimation of the effect sizes. The detailed risk of bias assessment for the domains is presented in Additional files 2.

**Fig. 7:**
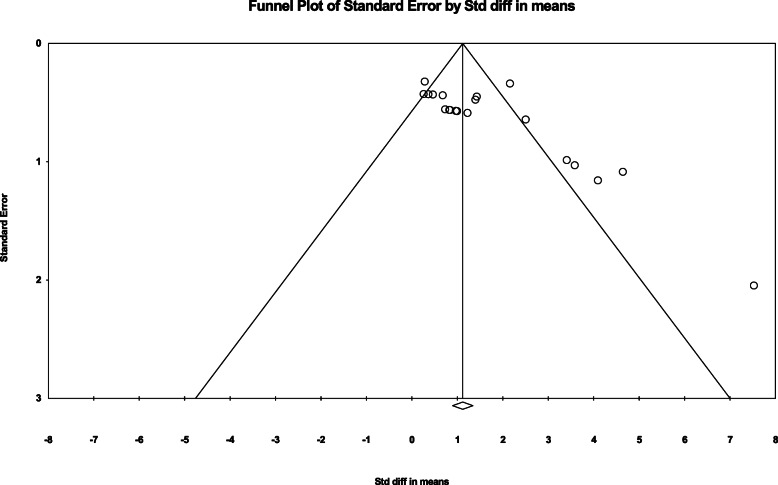
Funnel plot

Figure 7 is a funnel plot showing the relationship between the sample size and the effect size to confirm publication bias. We visually checked whether the funnel plot was symmetrical and checked Egger’s regression to determine statistically significant. Publication bias existed because the funnel plot was not symmetric and had a *p*-value of 0.01. However, without Garcia’s study [62], which had a high risk of bias, the funnel plot had a symmetrical structure and a *p*-value of 0.121, so it can be said that there was no publication bias. In the case of safety, since there were fewer than 10 documents included, the minimum conditions for publication bias were not satisfied, and the publication bias of the side effects was not analyzed.

## Discussion

The effect size of the therapeutic effect had an SMD of 1.394 (95% CI: 0.944–1.843), which was significant for treating depression [69] and except for Garcia’s study [62], in which reporting bias was suspected because of the selection reporting, the effect size SMD was 1.074, (95% CI: 0.689–1.459), which was also a meaningful result. The studies excluded by the exclusion criteria also showed positive treatment effects of rTMS for PPD, supporting the treatment effect. In Brock’s study, 14 out of 19 patients with rTMS achieved remission [70], and in Ozmut’s study, eight out of 15 patients responded to rTMS [71]. Other studies also succeeded in improving the depression of patients with PPD using rTMS [72, 73]. It could be possible that the effect size was overestimated because the parameters that can affect the treatment effect, such as the site of stimulation, frequency, and interval time, were not established, and there was a risk of bias. However, even considering these points, the treatment potential was confirmed, and if a protocol becomes established, it is expected that more sophisticated effect sizes can be calculated.

The effect size of TMS side effects had an SMD of 0.346 (95% CI: 0.214–0.506), which was significant [69]. However, the side effects of rTMS treatment on the mothers, such as headaches, discomfort, and pain in the stimulation area, were minor, and these side effects disappeared at the end of the treatment process. Some mothers experienced supine hypotension [60], but this was caused by a posture problem during treatment and could be prevented through postural correction [68].

All children born to mothers with rTMS treatment were born healthy. In one of the RCT studies that evaluated the child’s health condition by appearance-pulse-grimace-activity-respiration scores, the difference between the two groups was not significant [experimental group 8.36 (1.50), control group 8.73 (0.90), *p* = 0.501] [58]. There were five preterm births and one brachial plexus injury among the fetuses. Although 5% (5) of the study participants experienced preterm birth, two of these had already been warned of the risk of preterm birth by a biomedical test. Considering that the average proportion of women who undergo preterm birth worldwide is 11% [74], it is unlikely that a causal relationship exists between rTMS and preterm birth. However, further research on this is needed. Brachial plexus injury was reported as a side effect in one newborn, but it was not related to rTMS treatment [58], and there were no other side effects in the infants. One study that checked the child of a mother treated with rTMS reported that the exposure during pregnancy did not affect the cognitive or motor development of the child [75]. High frequencies above 40 Hz affect the lungs and immune system of the fetus [76, 77], and the maximum electromagnetic field applicable to the fetus is 800 mv/m [78]. However, the frequency used in rTMS treatment is generally 1–25 Hz, and the scale of the electromagnetic field is 100 mv/m [79]. Nevertheless, additional research should be conducted because there are many aspects not yet researched.

Mothers do not prefer the current treatment for peripartum depression. They are not willing to take antidepressants because they are concerned not only about the side effects that they will suffer but also the side effects to the fetus [80, 81] Besides, 2.5% of mothers suffer from treatment-resistant depression and have to choose a different treatment method [82]. In one survey on depression treatments, only 1.2% of the population accepted ECT treatment [83]. Although the acceptance rate of rTMS was also low, when knowledge about the treatment was shared, the acceptance rate increased by more than 50%, showing the possibility as a treatment method for PPD [84].

From the side of economic efficiency, rTMS may be a good alternative for treating PPD. For example, rTMS might save $112 per quality-adjusted life years (QALY) compared to antidepressants [85] and might save $8515 per year compared to ECTs [86]. rTMS could reduce anxiety about the cost, which was one of the risk factors that had the most significant impact on pregnant women [87]. Consequently, the socioeconomic costs of peripartum depression might be reduced.

### Limitation and future study direction

Regarding the limitations of the meta-analysis, our results showed high heterogeneity, and the reason may be heterogeneity due to methodological diversity because the parameters of rTMS varied from study to study, or heterogeneity due to coincidence because the analysis was conducted with small sample-sized studies. However, because the parameters vary according to the patient’s situation (e.g., the stimulation site changes according to symptoms and the frequency changes according to the stimulation site), it should be judged as clinical heterogeneity. The lack of patients and studies might also constrain our results. Thus, the effects of rTMS on PPD and should be interpreted with caution. Future research should focus on the effects in the prenatal and infancy periods and establish parameters for rTMS, such as the site stimulation or frequency.

## Conclusions

We collected data from existing documents and identified 10 studies that were suitable for a systematic review, of which five are eligible for meta-analysis. According to the analysis of the included studies, the therapeutic effect size of rTMS for PPD showed an SMD of 1.394 (95% CI: 0.944–1.843), and the sensitivity analysis showed an SMD = 1.074 (95% CI: 0.689–1.459), which is a significant effect, but this effect size could be overestimated because more than 50% of the studies were NRS, and some studies were at risk of bias Nonetheless, rTMS seems to have potential as a treatment for peripartum depression. The side effects of rTMS for PPD had an SMD of 0.346 (95% CI: 0.214–0.506), which is meaningful. However, there were no serious side effects to the mothers or fetuses. From a variety of perspectives, the treatment of PPD using rTMS could be considered an alternative treatment to avoid exposure of the fetus to drugs and the severe side effects of ECT. Further research is required to increase the confidence in these results.

## Supplementary Information

**Additional file 1: Appendix 1.** Excluded literature and the reason.

**Additional file 2: Appendix 2.** Risk assessment of selected literature.

## Data Availability

No data or materials were generated for this narrative review.

## Abbreviations

PPD: Peripartum depression
MDD: Major depressive disorder
ECT: Electroconvulsive therapy
MAO: Monoamine oxidase
rTMS: Repeated transcranial magnetic stimulation
DLPFC: Dorsolateral prefrontal cortex
RCT: Randomized controlled trial
NRS: Non-randomized studies
DSM-5: Diagnostic and Statistical Manual of Mental Disorders-5
HDRS: Hamilton Depression Rating Scale
ROB2: Risk of bias 2
ROBINS-I: The Risk of Bias Assessment tool for Nonrandomized Studies
SMD: Standardized Mean Deviation
CI: Confidence interval
MARDS: Montgomery-Åsberg Depression Rating Scale
BDI: Beck Depression Inventory.
EPDS: Edinburgh Postnatal Depression Scale
QALY: Quality adjusted life year
CGI-S: Clinical Global impression scale
GAS: Global Assessment Scale
SF-36-V: 36-item Quality of Life Health Survey Vitality scores
SF-36-MH: 36-item Quality of Life Health Survey Mental Health scores
