# Supplementary material for: Repetitive transcranial magnetic stimulation treatment for peripartum depression: systematic review & meta-analysis
Source: BMC Pregnancy Childbirth. 2021 Feb 9;21:118. doi: 10.1186/s12884-021-03600-3 (PMC7874443; doi:10.1186/s12884-021-03600-3)
Supplement: Supplementary file 1 — Additional file 1: Appendix 1. Excluded literature and the reason. [file 12884_2021_3600_MOESM1_ESM.docx]

Appendix 2: Excluded studies

| **Studies** | **Exclude reason** |
| --- | --- |
| (Nahas et al., 1999) [1] | Specific therapeutic effect doesn’t mention. |
| (Gonsalves et al., 2006) [2] | Missing data. |
| (Kim et al., 2009) [3] | no clinical outcome. |
| (Tjoa et al., 2010) [4] | No outcome and no protocol |
| (Andriotti et al., 2017) [5] | no clinical outcome. |
| (Epstein et al., 2014) [6] | no clinical outcome |
| (Bhola et al., 2015) [7] | no clinical outcome |
| (Eryilmaz et al., 2015) [8] | no protocol |
| (Ozmut et al., 2015) [9] | Only abstract. |
| (Brock et al., 2016) [10] | Only abstract. |
| (Ebbing, 2019) [11] | Missing data |
| (Stultz et al., 2018) [12] | Only abstract |
| (Trevizol et al., 2019) [13] | Using Intermittent theta burst stimulation |
| ( Konstantinou GN., 2020) [14] | review |
| ( Ganho-Ávila A., 2019) [15] | review |
| ( Felipe RdM., 2016) [16] | review |
| ( Cole J.et al, 2019) [17] | review |
| (Burton.et al, 2014) [18] | Inappropriate indication (bipolar depression) |
| (Xiong.et al, 2018) [19] | Inappropriate indication (bipolar depression) |
| (Tarhan et al, 2012) [20] | no clinical outcome |

1. Nahas Z, Bohning D, Molloy A, Oustz JA, Risch S, George M: **Safety and Feasibility of Repetitive Transcranial Magnetic Stimulation in the Treatment of Anxious Depression in Pregnancy**. *The Journal of clinical psychiatry* 1999, **60**:50-52.

2. Gonsalves L, Schuermeyer I: **Treating depression in pregnancy: practical suggestions**. *Cleveland Clinic journal of medicine* 2006, **73**(12):1098.

3. Kim D, Gonzalez J, O'Reardon J: **Pregnancy and Depression: Exploring a New Potential Treatment Option**. *Current psychiatry reports* 2009, **11**:443-446.

4. Tjoa C, Pare E, Kim DR: **Unipolar Depression During Pregnancy: Nonpharmacologic Treatment Options**. *Women's Health* 2010, **6**(4):565-576.

5. Andriotti T, Stavale R, Nafee T, Fakhry S, Mohamed MMA, Sofiyeva N, Ganho-Avila A, Bogner A, Barbosa SP, Piton LS *et al*: **ASSERT trial - How to assess the safety and efficacy of a high frequency rTMS in postpartum depression ? A multicenter, double blinded, randomized, placebo-controlled clinical trial**. *Contemp Clin Trials Commun* 2017, **5**:86-91.

6. Epstein RA, Moore KM, Bobo WV: **Treatment of nonpsychotic major depression during pregnancy: patient safety and challenges**. *Drug Healthc Patient Saf* 2014, **6**:109-129.

7. Bhola R, Kinsella E, Giffin N, Lipscombe S, Ahmed F, Weatherall M, Goadsby PJ: **Single-pulse transcranial magnetic stimulation (sTMS) for the acute treatment of migraine: evaluation of outcome data for the UK post market pilot program**. *J Headache Pain* 2015, **16**:535.

8. Eryilmaz G, Sayar GH, Ozten E, Gul IG, Yorbik O, Isiten N, Bagci E: **Follow-up study of children whose mothers were treated with transcranial magnetic stimulation during pregnancy: preliminary results**. *Neuromodulation* 2015, **18**(4):255-260.

9. Ozmut O, Balibey H, Yilan Y, Algul A, Ebrinc S, Cetin M, Tutuncu R, Ates A, Basoglu C: **Repetitive transcranial magnetic stimulation for the treatment of depression during pregnancy and postpartum period**. *Bulletin of Clinical Psychopharmacology* 2015, **25**(1):S203-S204.

10. Brock DG, Demitrack MA, Groom P, Holbert R, Rado JT, Gross PK, Goethe JW, Schrodt GR, Weeks HR: **Effectiveness of NeuroStar transcranial magnetic stimulation (TMS) in patients with major depressive disorder with postpartum onset**. *Brain Stimulation* 2016, **9**(5).

11. Ebbing J, van de Lindt D: **Right sided (RDLPFC) low frequency (1Hz) rTMS in the third trimester of pregnancy; a case report**. *Brain Stimulation: Basic, Translational, and Clinical Research in Neuromodulation* 2019, **12**(2):533.

12. Stultz DJ, Thistlethwaite D, Voltin R, Osburn S, Walton R, Burns T: **Bipolar, depressed, and pregnant – transcranial magnetic stimulation as a treatment alternative**. *Brain Stimulation* 2018, **11**(6).

13. Trevizol AP, Vigod SN, Daskalakis ZJ, Vila-Rodriguez F, Downar J, Blumberger DM: **Intermittent theta burst stimulation for major depression during pregnancy**. *Brain Stimul* 2019, **12**(3):772-774.

14. Konstantinou GN, Vigod SN, Mehta S, Daskalakis ZJ, Blumberger DM: **“A systematic review of non-invasive neurostimulation for the treatment of depression during pregnancy”**. *Journal of Affective Disorders* 2020, **272**:259-268.

15. Ganho-Ávila A, Poleszczyk A, Mohamed MMA, Osório A: **Efficacy of rTMS in decreasing postnatal depression symptoms: A systematic review**. *Psychiatry Res* 2019, **279**:315-322.

16. Felipe RdM, Ferrão YA: **Transcranial magnetic stimulation for treatment of major depression during pregnancy: a review**. *Trends in Psychiatry and Psychotherapy* 2016, **38**:190-197.

17. Cole J, Bright K, Gagnon L, McGirr A: **A systematic review of the safety and effectiveness of repetitive transcranial magnetic stimulation in the treatment of peripartum depression**. *Journal of Psychiatric Research* 2019, **115**:142-150.

18. Burton C, Gill S, Clarke P, Galletly C: **Maintaining remission of depression with repetitive transcranial magnetic stimulation during pregnancy: a case report**. *Arch Womens Ment Health* 2014, **17**(3):247-250.

19. Xiong W, Lopez R, Cristancho P: **Transcranial magnetic stimulation in the treatment of peripartum bipolar depression: a case report**. *Braz J Psychiatry* 2018, **40**(3):344-345.

20. Tarhan N, Sayar FG, Tan O, Kagan G: **Efficacy of high-frequency repetitive transcranial magnetic stimulation in treatment-resistant depression**. *Clin EEG Neurosci* 2012, **43**(4):279-284.
