# Supplementary material for: Repetitive transcranial magnetic stimulation treatment for peripartum depression: systematic review & meta-analysis
Source: BMC Pregnancy Childbirth. 2021 Feb 9;21:118. doi: 10.1186/s12884-021-03600-3 (PMC7874443; doi:10.1186/s12884-021-03600-3)
Supplement: Supplementary file 2 — Additional file 2: Appendix 2. Risk assessment of selected literature. [file 12884_2021_3600_MOESM2_ESM.docx]

Appendix 3 Risk of bias assessment [RCT]

Revised Cochrane risk-of-bias tool for randomized trials (RoB 2)

Response options Y: yes, PY: probably yes, N: no, PN: probably no, NI: no information

Risk of bias assessment

Randomized controlled trial of transcranial magnetic stimulation in pregnant women with major depressive disorder - Kim et al 2019

Domain 1: Risk of bias arising from the randomization

| Signaling questions | Comments | Response options |
| --- | --- | --- |
| 1.1 Was the allocation sequence random? | Study mentioned use randomization but detailed of randomization was not mentioned. | No information |
| 1.2 Was the allocation sequence concealed until participants were enrolled and assigned to interventions? | Study mentioned their coils used in study had identical physical appearances.” | Yes |
| 1.3 Did baseline differences between intervention groups suggest a problem with the randomization process? | Study mentioned that “active treatment and sham treatment were statistically similar, p=0.246” | No |
| Risk-of-bias judgement | Q 1.2 response was Yes -> Q 1.1 response was NI* ->  Q 1.3 response was No -> Low risk | Low risk |

Domain 2: Risk of bias due to deviations from the intended interventions (effect of assignment to intervention)

| Signaling questions | Comments | Response options |
| --- | --- | --- |
| 2.1. Were participants aware of their assigned intervention during the trial? | The subject, were blinded to the randomization assignment and study tested blind and result was that sham system was an effective. | No |
| 2.2. Were carers and people delivering the interventions aware of participants' assigned intervention during the trial? | TMS administrator, and rater were blinded to the randomization assignment and study tested blind and result was that sham system was an effective. | No |
| 2.3. Were there deviations from the intended intervention that arose because of the trial context? | No response because 2.2 response is not Y/PY*/NI. | * |
| 2.4 Were these deviations likely to have affected the outcome? | No response because 2.3 response is not Y/PY. | * |
| 2.5. Were these deviations from intended intervention balanced between groups? | No response because 2.5 response is not Y/PY/NI. | * |
| 2.6 Was an appropriate analysis used to estimate the effect of assignment to intervention? | The integrity of the blind test was performed in 10 participants naive to TMS exposure but participants felt almost of them receiving the active coil and there were no significant differences in their responses by coil. | Yes |
| 2.7 Was there potential for a substantial impact (on the result) of the failure to analyze participants in the group to which they were randomized? | No response because 2.6 response is not N/PN/NI. | * |
| Risk-of-bias judgement | Q 2.1 response was No -> Q 2.2 response was NO  Q 2.6 response was Yes -> Low risk | Low risk |

Domain 3: Missing outcome data

| Signaling questions | Comments | Response options |
| --- | --- | --- |
| 3.1 Were data for this outcome available for all, or nearly all, participants randomized? | 2 patients did not complete full sessions without reason.  study just mentioned “no subjects were withdrawn by investigators”  but Nearly all data (over the 90 % data) was available | Probably Yes |
| 3.2 Is there evidence that the result was not biased by missing outcome data? | No response because 3.1 response is not N/PN/NI. | * |
| 3.3 Could missingness in the outcome depend on its true value? | No response because 3.2 response is not N/PN. | * |
| 3.4 Is it likely that missingness in the outcome depended on its true value? | No response because 3.2 response is not Y/PY/NI. | * |
| Risk-of-bias judgement | Q 3.1 response was Yes -> Low risk | Low risk |

Domain 4: Risk of bias in measurement of the outcome

| Signaling questions | Comments | Response options |
| --- | --- | --- |
| 4.1 Was the method of measuring the outcome inappropriate? | Outcome measuring method was appropriate to confirm scale of depression | No |
| 4.2 Could measurement or ascertainment of the outcome have differed between intervention groups? | Between intervention groups, measurement or ascertainment of the outcome was not different | No |
| 4.3 Were outcome assessors aware of the intervention received by study participants? | Study mentioned “outcome assessors were blinded to the randomization assignment.” | No |
| 4.4 Could assessment of the outcome have been influenced by knowledge of intervention received? | No Response because 4.3 response is not Y/PY/NI. | * |
| 4.5 Is it likely that assessment of the outcome was influenced by knowledge of intervention received? | No Response because 4.4 response is not Y/PY/NI. | * |
| Risk-of-bias judgement | Q 4.1 response was No -> Q 4.2 response was No ->  Q 4.3 response was No -> Low risk | Low risk |

Domain 5: Risk of bias in selection of the reported result

| Signaling questions | Comments | Response options |
| --- | --- | --- |
| 5.1 Were the data that produced this result analyzed in accordance with a pre-specified analysis plan that was finalized before unblinded outcome data were available for analysis? | The method of outcome was reported on trial plan. | Yes |
| Is the numerical result being assessed likely to have been selected, on the basis of the results, from multiple eligible outcome measurements (e.g. scales, definitions, time points) within the outcome domain? | The study use multiple outcome measurements but all measurements were reported and planned before. | No |
| Is the numerical result being assessed likely to have been selected, on the basis of the results, from multiple eligible analyses of the data? | No multiple eligible analysis use ITT (Intention-to-treat) | No |
| Risk-of-bias judgement | Q 5.2 response was No -> Q 5.3 response was No ->  Q 5.1 response was Yes -> Low risk | Low risk |

Overall risk of bias

| Risk-of-bias judgement | All domain has Low risk, therefore overall risk of bias is low | Low risk |
| --- | --- | --- |

Effects of repetitive transcranial magnetic stimulation on clinical, social, and cognitive performance in postpartum depression –Myczkowski ML et al, 2012

Domain 1: Risk of bias arising from the randomization

| Signaling questions | Comments | Response options |
| --- | --- | --- |
| 1.1 Was the allocation sequence random? | The study used computerized random-number generator according to CONSORT guidelines. | Yes |
| 1.2 Was the allocation sequence concealed until participants were enrolled and assigned to interventions? | The detailed about concealed was not reported in study but  the study followed CONSORT guideline which recommended allocating sequence concealment. | Probably Yes |
| 1.3 Did baseline differences between intervention groups suggest a problem with the randomization process? | Between two groups did not show results significantly difference | No |
| Risk-of-bias judgement | Q 1.2 response was Yes -> Q 1.1 response was Probably Yes ->  Q 1.3 response was No -> Low risk | Low risk |

Domain 2: Risk of bias due to deviations from the intended interventions (effect of assignment to intervention)

| Signaling questions | Comments | Response options |
| --- | --- | --- |
| 2.1. Were participants aware of their assigned intervention during the trial? | In this study, Because of Using Sham intervention, participants did not aware of their assigned intervention. | No |
| 2.2. Were carers and people delivering the interventions aware of participants' assigned intervention during the trial? | There is no information about carers and people delivering the interventions. | No information |
| 2.3. Were there deviations from the intended intervention that arose because of the trial context? | There were no deviations because of the trial context | No |
| 2.4 Were these deviations likely to have affected the outcome? | No Response because 2.3 response is not Y/PY. | * |
| 2.5. Were these deviations from intended intervention balanced between groups? | No Response because 2.4 response is not Y/PY/NI. | * |
| 2.6 Was an appropriate analysis used to estimate the effect of assignment to intervention? | 60% of patients guessed correctly when asked about which group they believed they had seen assigned to. Using ITT(Intendtion-to-treat) method | No |
| 2.7 Was there potential for a substantial impact (on the result) of the failure to analyze participants in the group to which they were randomized? | No Response because 2.6 response is not N/PN/NI. | * |
| Risk-of-bias judgement | Q 2.1 response was No -> Q 2.2 response was Probably NI ->  Q 2.3 response was No -> Q 2.6 response was No -> Low risk | Low risk |

Domain 3: Missing outcome data

| Signaling questions | Comments | Response options |
| --- | --- | --- |
| 3.1 Were data for this outcome available for all, or nearly all, participants randomized? | All participants completed the study | Yes |
| 3.2 Is there evidence that the result was not biased by missing outcome data? | No Response because 3.1 response is not N/PN/NI. | * |
| 3.3 Could missingness in the outcome depend on its true value? | No Response because 3.2 response is not N/PN. | * |
| 3.4 Is it likely that missingness in the outcome depended on its true value? | No Response because 3.3 response is not Y/PY/NI. | * |
| Risk-of-bias judgement | Q 3.1 response was Yes -> Low risk | Low risk |

Domain 4: Risk of bias in measurement of the outcome

| Signaling questions | Comments | Response options |
| --- | --- | --- |
| 4.1 Was the method of measuring the outcome inappropriate? | Outcome measuring method was appropriate to confirm scale of depression | No |
| 4.2 Could measurement or ascertainment of the outcome have differed between intervention groups? | Between intervention groups, measurement or ascertainment of the outcome was not different. | No |
| 4.3 Were outcome assessors aware of the intervention received by study participants? | The rater guessed the assignment correctly in 54% of cases. | No |
| 4.4 Could assessment of the outcome have been influenced by knowledge of intervention received? | No Response because 4.3 response is not Y/PY/NI. | * |
| 4.5 Is it likely that assessment of the outcome was influenced by knowledge of intervention received? | No Response because 4.4 response is not Y/PY/NI. | * |
| Risk-of-bias judgement | Q 4.1 response was No -> Q 4.2 response was No ->  Q 4.3 response was No -> Low risk | Low risk |

Domain 5: Risk of bias in selection of the reported result

| Signaling questions | Comments | Response options |
| --- | --- | --- |
| 5.1 Were the data that produced this result analyzed in accordance with a pre-specified analysis plan that was finalized before unblinded outcome data were available for analysis? | No Response because 5.2 response is not N/PN. | * |
| Is the numerical result being assessed likely to have been selected, on the basis of the results, from multiple eligible outcome measurements (e.g. scales, definitions, time points) within the outcome domain? | The study use multiple outcome measurements and some measurements were not reported on a plan (CGI-2 missing) | Yes |
| Is the numerical result being assessed likely to have been selected, on the basis of the results, from multiple eligible analyses of the data? | The study use multiple analyses but all measurements were reported and planned beforehand | No |
| Risk-of-bias judgement | Q 5.2 response was Yes -> Q 5.3 response was No -> Some concerns | Some concerns |

Overall risk of bias

| Risk-of-bias judgement | Because one domain was Some concerns,  overall risk of bias is some concerns. | Some concerns |
| --- | --- | --- |

Risk of bias assessment [NRS]

Revised Cochrane a risk of bias tool to assess non-randomized studies of interventions

An open label pilot study of transcranial magnetic stimulation for pregnant women with major depressive disorder - Kim et al 2013

Domain 1: Risk of bias due to confounding

| Signaling questions | Comments | Response options |
| --- | --- | --- |
| 1.1 Is there potential for confounding of the effect of intervention in this study? | 4 patients take an antidepressant. Although no changes were made in antidepressant dosing for at least 2 weeks before study entry, it could be affect to confirm effect of rTMS. | Probably Yes |
| 1.2. Was the analysis based on splitting participants’ follow up time according to intervention received? | Study did not base on splitting participants’ follow up time. | Probably No |
| 1.3. Were intervention discontinuations or switches likely to be related to factors that are prognostic for the outcome? | No response because 1.2 response is not Y/PY. | * |
| 1.4. Did the authors use an appropriate analysis method that controlled for all the important confounding domains? | Study reported not only Intention-to-treat analysis but also analysis considering whether or not to take antidepressant. | No |
| 1.5. Were confounding domains that were controlled for measured validly and reliably by the variables available in this study? | No response because 1.4 response is not Y/PY. | * |
| 1.6. Did the authors control for any post-intervention variables that could have been affected by the intervention? | Authors considered antidepressants. the participants who take antidepressants were checking dose of antidepressants and only participants who had no changes dosing for at least 2 weeks before study entry could participate trial. | Yes |
| 1.7. Did the authors use an appropriate analysis method that controlled for all the important confounding domains and for time-varying confounding? | No response because 1.3 response is not Y/PY. | * |
| 1.8. Were confounding domains that were controlled for measured validly and reliably by the variables available in this study? | No response because 1.7 response is not Y/PY | * |
| Risk of bias judgement | Antidepressants could be confounding factor but confounding domains are appropriately measured and controlled. | Moderate risk |

Domain 2: Risk of bias in selection of participants into the study

| Signaling questions | Comments | Response options |
| --- | --- | --- |
| 2.1. Was selection of participants into the study (or into the analysis) based on participant characteristics observed after the start of intervention? | Participants of study selected before the start of intervention. | No |
| 2.2. Were the post-intervention variables that influenced selection likely to be associated with intervention? | No response because 2.1 response is not Y/PY. | * |
| 2.3 Were the post-intervention variables that influenced selection likely to be influenced by the outcome or a cause of the outcome? | No response because 2.2 response is not Y/PY. | * |
| 2.4. Do start of follow-up and start of intervention coincide for most participants? | Start of follow up and start of intervention coincided for most participants | Yes |
| 2.5. Were adjustment techniques used that are likely to correct for the presence of selection biases? | No response because 2.2 response is not Y/PY or 2.4 response is not N/PN. | * |
| Risk of bias judgement | All participants who would have been eligible for the target trial were included in the study; | Low risk |

Domain 3: Risk of bias in classification of interventions

| Signaling questions | Comments | Response options |
| --- | --- | --- |
| 3.1 Were intervention groups clearly defined? | Eligible criteria are clearly defined. For example, 18-39 years old and 14-34 pregnancy period with a DSM-IV diagnosis of MDD based on a counseling. and Satisfy Clinical Global Impression Severity scale > 3 and Hamilton Depression Rating Scale score > 13. | Probably Yes |
| 3.2 Was the information used to define intervention groups recorded at the start of the intervention? | At the start of the intervention, intervention group were defined | Yes |
| 3.3 Could classification of intervention status have been affected by knowledge of the outcome or risk of the outcome? | Knowledge about outcome or risk of the outcome had not been affected classification of intervention status. | No |
| Risk of bias judgement | Intervention status is well defined. | Low risk |

Domain 4: Risk of bias due to deviations from intended interventions

| Signaling questions | Comments | Response options |
| --- | --- | --- |
| 4.1 Were there deviations from the intended intervention beyond what would be expected in usual practice? | No response because aim for this study is to assess the effect of starting and adhering to intervention. | * |
| 4.2 Were these deviations from intended intervention unbalanced between groups and likely to have affected the outcome? | No response because aim for this study is to assess the effect of starting and adhering to intervention. | * |
| 4.3. Were important co-interventions balanced across intervention groups? | Co-interventions were balanced across intervention group. | Probably Yes |
| 4.4. Was the intervention implemented successfully for most participants? | Yes. most participants was implemented successfully (100%) | Yes |
| 4.5. Did study participants adhere to the assigned intervention regimen? | Yes. All participants adhere assigned intervention regimen | Yes |
| 4.6. Was an appropriate analysis used to estimate the effect of starting and adhering to the intervention? | No response because response 4.3, 4.4 or 4.5:response is not N/PN. | * |
| Risk of bias judgement | The important co-interventions were balanced across interventions group | Low risk |

Domain 5: Risk of bias due to missing data

| Signaling questions | Comments | Response options |
| --- | --- | --- |
| 5.1 Were outcome data available for all, or nearly all, participants? | Outcome data of nearly all participants was available. | Yes |
| 5.2 Were participants excluded due to missing data on intervention status? | 3 participants were excluded but they were excluded screening before start of interventions and all participants completed full sessions. | No |
| 5.3 Were participants excluded due to missing data on other variables needed for the analysis? | All participants completed full sessions. | No |
| 5.4 Are the proportion of participants and reasons for missing data similar across interventions? | No response because 5.1 response is not N/PN or 5.2 & 5.3 response is not Y/PY. | * |
| 5.5 Is there evidence that results were robust to the presence of missing data? | No response because 5.1 response is not N/PN or 5.2 & 5.3 response is not Y/PY | * |
| Risk of bias judgement | Data were all complete | Low risk |

Domain 6: Risk of bias in measurement of outcomes

| Signaling questions | Comments | Response options |
| --- | --- | --- |
| 6.1 Could the outcome measure have been influenced by knowledge of the intervention received? | Because of study design is open label, Knowledge of the intervention received could influence outcome measure. | Probably Yes |
| 6.2 Were outcome assessors aware of the intervention received by study participants? | This study did not any blinding procedure. | Yes |
| 6.3 Were the methods of outcome assessment comparable across intervention groups? | Outcome assessment is comparable across before and after. | Yes |
| 6.4 Were any systematic errors in measurement of the outcome related to intervention received? | There are not systematic errors in measurement of the outcome related to intervention received. | No |
| Risk of bias judgement | Outcome measurement were comparable but it could be influenced by knowledge of the intervention received by study participants | Moderate risk |

Domain 7: Risk of Bias in selection of the reported result

| Signaling questions | Comments | Response options |
| --- | --- | --- |
| 7.1.Is the reported effect estimate likely to be selected, on the basis of the results from multiple outcome measurements within the outcome domain? | Study did not reported selectively and the pre-planning and the results are consistent. | No |
| 7.2 Is the reported effect estimate likely to be selected, on the basis of the results from multiple analyses of the intervention-outcome relationship? | Study did not report selectively and the pre-planning and the results are consistent. | No |
| 7.3 Is the reported effect estimate likely to be selected, on the basis of the results from different subgroups? | Study did not report selectively and the pre-planning and the results are consistent. | No |
| Risk of bias judgement | The study was well-performed through examination of a pre-registered protocol and statistical analysis plan. | Low risk |

Overall risk of bias

| Risk-of-bias judgement | Because risk of bias due to confounding domain are moderate risk, overall risk of bias is moderate. | Moderate risk |
| --- | --- | --- |

Transcranial magnetic stimulation during pregnancy - Hizli Sayar G et al, 2014

Domain 1: Risk of bias due to confounding

| Signaling questions | Comments | Response options |
| --- | --- | --- |
| 1.1 Is there potential for confounding of the effect of intervention in this study? | Because all patients were treatment-resistant, there is no need to consider antidepressants. | No |
| 1.2. Was the analysis based on splitting participants’ follow up time according to intervention received? | No response because 1.1 response is not Y/PY. | * |
| 1.3. Were intervention discontinuations or switches likely to be related to factors that are prognostic for the outcome? | No response because 1.2 response is not Y/PY. | * |
| 1.4. Did the authors use an appropriate analysis method that controlled for all the important confounding domains? | No response because 1.3 response is not N/PN. | * |
| 1.5. Were confounding domains that were controlled for measured validly and reliably by the variables available in this study? | No response because 1.4 response is not Y/PY. | * |
| 1.6. Did the authors control for any post-intervention variables that could have been affected by the intervention? | No response because 1.3 response is not N/PN. | * |
| 1.7. Did the authors use an appropriate analysis method that controlled for all the important confounding domains and for time-varying confounding? | No response because 1.3 response is not Y/PY. | * |
| 1.8. Were confounding domains that were controlled for measured validly and reliably by the variables available in this study? | No response because 1.7 response is not Y/PY. | * |
| Risk of bias judgement | It is thought that no confounding is predicted in this study. | Low risk |

Domain 2: Risk of bias in selection of participants into the study

| Signaling questions | Comments | Response options |
| --- | --- | --- |
| 2.1. Was selection of participants into the study (or into the analysis) based on participant characteristics observed after the start of intervention? | Participants of study selected before the start of intervention. | No |
| 2.2. Were the post-intervention variables that influenced selection likely to be associated with intervention? | No response because 2.1 response is not Y/PY. | * |
| 2.3 Were the post-intervention variables that influenced selection likely to be influenced by the outcome or a cause of the outcome? | No response because 2.2 response is not Y/PY. | * |
| 2.4. Do start of follow-up and start of intervention coincide for most participants? | Start of follow up and start of intervention coincided for most participants | Yes |
| 2.5. Were adjustment techniques used that are likely to correct for the presence of selection biases? | No response because 2.2 response is not Y/PY or 2.4 response is not N/PN. | * |
| Risk of bias judgement | All participants who would have been eligible for the target trial were included in the study; | Low risk |

Domain 3: Risk of bias in classification of interventions

| Signaling questions | Comments | Response options |
| --- | --- | --- |
| 3.1 Were intervention groups clearly defined? | Study have a strict screening process about participants | Yes |
| 3.2 Was the information used to define intervention groups recorded at the start of the intervention? | At the start of the intervention, intervention group were defined | Yes |
| 3.3 Could classification of intervention status have been affected by knowledge of the outcome or risk of the outcome? | Knowledge about outcome or risk of the outcome had not been affected classification of intervention status. | No |
| Risk of bias judgement | Intervention status is well defined. | Low risk |

Domain 4: Risk of bias due to deviations from intended interventions

| Signaling questions | Comments | Response options |
| --- | --- | --- |
| 4.1 Were there deviations from the intended intervention beyond what would be expected in usual practice? | No response because aim for this study is to assess the effect of starting and adhering to intervention. | * |
| 4.2 Were these deviations from intended intervention unbalanced between groups and likely to have affected the outcome? | No response because aim for this study is to assess the effect of starting and adhering to intervention. | * |
| 4.3. Were important co-interventions balanced across intervention groups? | Co-interventions were balanced across intervention group. | Probably Yes |
| 4.4. Was the intervention implemented successfully for most participants? | Yes most participants was implemented successfully (96.6%) | Yes |
| 4.5. Did study participants adhere to the assigned intervention regimen? | Yes All participants adhere assigned intervention regimen | Yes |
| 4.6. Was an appropriate analysis used to estimate the effect of starting and adhering to the intervention? | No response because response 4.3, 4.4 or 4.5:response is not N/PN. | * |
| Risk of bias judgement | The important co-interventions were balanced across interventions group | Low risk |

Domain 5: Risk of bias due to missing data

| Signaling questions | Comments | Response options |
| --- | --- | --- |
| 5.1 Were outcome data available for all, or nearly all, participants? | Outcome data of nearly all participants was available. | Probably Yes |
| 5.2 Were participants excluded due to missing data on intervention status? | 1 participant were excluded and that reason for withdrawal was reconsideration of the risks and benefits of the study. However it should be considered because the number of samples is small. | Probably Yes |
| 5.3 Were participants excluded due to missing data on other variables needed for the analysis? | All participants completed full sessions. | No |
| 5.4 Are the proportion of participants and reasons for missing data similar across interventions? | No response because 5.1 response is not N/PN or 5.2 & 5.3 response is not Y/PY | * |
| 5.5 Is there evidence that results were robust to the presence of missing data? | No response because 5.1 response is not N/PN or 5.2 & 5.3 response is not Y/PY | * |
| Risk of bias judgement | 96.6% data was available and this scale is enough to confirm effects of rTMS. | Low risk |

Domain 6: Risk of bias in measurement of outcomes

| Signaling questions | Comments | Response options |
| --- | --- | --- |
| 6.1 Could the outcome measure have been influenced by knowledge of the intervention received? | Knowledge of the intervention received could influence outcome measure. | Probably Yes |
| 6.2 Were outcome assessors aware of the intervention received by study participants? | This study did not mentioned about blinding | No information |
| 6.3 Were the methods of outcome assessment comparable across intervention groups? | Outcome assessment is comparable across before and after. | Yes |
| 6.4 Were any systematic errors in measurement of the outcome related to intervention received? | There are not systematic errors in measurement of the outcome related to intervention received. | No |
| Risk of bias judgement | Outcome measurement were comparable but it could be influenced by knowledge of the intervention received by study participants and no information about blinding | Moderate risk |

Domain 7: Risk of Bias in selection of the reported result

| Signaling questions | Comments | Response options |
| --- | --- | --- |
| 7.1.Is the reported effect estimate likely to be selected, on the basis of the results from multiple outcome measurements within the outcome domain? | Study did not report selectively and the pre-planning and the results are consistent. | No |
| 7.2 Is the reported effect estimate likely to be selected, on the basis of the results from multiple analyses of the intervention-outcome relationship? | Study did not report selectively and the pre-planning and the results are consistent. | No |
| 7.3 Is the reported effect estimate likely to be selected, on the basis of the results from different subgroups? | Study did not report selectively and the pre-planning and the results are consistent. | No |
| Risk of bias judgement | The study was well-performed through examination of a pre-registered protocol and statistical analysis plan. | Low risk |

Overall risk of bias

| Risk-of-bias judgement | Because Risk of bias in measurement of outcomes domain are moderate risk, overall risk of bias is moderate | Moderate risk |
| --- | --- | --- |

Repetitive transcranial magnetic stimulation treats postpartum depression - Keith S. Garcia et al, 2012

Domain 1: Risk of bias due to confounding

| Signaling questions | Comments | Response options |
| --- | --- | --- |
| 1.1 Is there potential for confounding of the effect of intervention in this study? | Participants are antidepressant-free women and did not conducted simultaneous treatment. | Probably No |
| 1.2. Was the analysis based on splitting participants’ follow up time according to intervention received? | No response because 1.1 response is not Y/PY. | * |
| 1.3. Were intervention discontinuations or switches likely to be related to factors that are prognostic for the outcome? | No response because 1.2 response is not Y/PY. | * |
| 1.4. Did the authors use an appropriate analysis method that controlled for all the important confounding domains? | No response because 1.3 response is not N/PN. | * |
| 1.5. Were confounding domains that were controlled for measured validly and reliably by the variables available in this study? | No response because 1.4 response is not Y/PY. | * |
| 1.6. Did the authors control for any post-intervention variables that could have been affected by the intervention? | No response because 1.3 response is not N/PN. | * |
| 1.7. Did the authors use an appropriate analysis method that controlled for all the important confounding domains and for time-varying confounding? | No response because 1.3 response is not N/PN. | * |
| 1.8. Were confounding domains that were controlled for measured validly and reliably by the variables available in this study? | No response because 1.7 response is not Y/PY. | * |
| Risk of bias judgement | It is thought that no confounding is predicted in this study. | Low risk |

Domain 2: Risk of bias in selection of participants into the study

| Signaling questions | Comments | Response options |
| --- | --- | --- |
| 2.1. Was selection of participants into the study (or into the analysis) based on participant characteristics observed after the start of intervention? | Patients were selected before trial started | No |
| 2.2. Were the post-intervention variables that influenced selection likely to be associated with intervention? | No response because 2.1 response is not Y/PY. | * |
| 2.3 Were the post-intervention variables that influenced selection likely to be influenced by the outcome or a cause of the outcome? | No response because 2.2 response is not Y/PY. | * |
| 2.4. Do start of follow-up and start of intervention coincide for most participants? | Most participants did not coincide follow up and start of intervention | No |
| 2.5. Were adjustment techniques used that are likely to correct for the presence of selection biases? | In this study, no adjustment techniques were found to correct for the presence of selection biases | No |
| Risk of bias judgement | Start of follow up and start of intervention do not coincide for most participants but almost of them were selected before trial started. | Moderate risk |

Domain 3: Risk of bias in classification of interventions

| Signaling questions | Comments | Response options |
| --- | --- | --- |
| 3.1 Were intervention groups clearly defined? | Intervention groups were clearly defined. For example, 18 - 50 years old who had experienced an uncomplicated pregnancy and delivery that resulted in a healthy, single infant. and meeting DSM-IV criteria for a major depressive episode. | Yes |
| 3.2 Was the information used to define intervention groups recorded at the start of the intervention? | At the start of the intervention, intervention group were defined | Yes |
| 3.3 Could classification of intervention status have been affected by knowledge of the outcome or risk of the outcome? | Knowledge about outcome or risk of the outcome had not been affected classification of intervention status. | No |
| Risk of bias judgement | Intervention status is well defined. | Low risk |

Domain 4: Risk of bias due to deviations from intended interventions

| Signaling questions | Comments | Response options |
| --- | --- | --- |
| 4.1 Were there deviations from the intended intervention beyond what would be expected in usual practice? | No response because aim for this study is to assess the effect of starting and adhering to intervention. | * |
| 4.2 Were these deviations from intended intervention unbalanced between groups and likely to have affected the outcome? | No response because aim for this study is to assess the effect of starting and adhering to intervention. | * |
| 4.3. Were important co-interventions balanced across intervention groups? | Co-interventions were balanced across intervention group. | Probably Yes |
| 4.4. Was the intervention implemented successfully for most participants? | Yes most participants was implemented successfully (100%) | Yes |
| 4.5. Did study participants adhere to the assigned intervention regimen? | Yes All participants adhere assigned intervention regimen | Probably Yes |
| 4.6. Was an appropriate analysis used to estimate the effect of starting and adhering to the intervention? | No response because response 4.3, 4.4 or 4.5:response is not N/PN. | * |
| Risk of bias judgement | The important co-interventions were balanced across interventions group | Low risk |

Domain 5: Risk of bias due to missing data

| Signaling questions | Comments | Response options |
| --- | --- | --- |
| 5.1 Were outcome data available for all, or nearly all, participants? | Outcome data of two participants were unavailable but total participants are only 9. it could be considered. | Probably No |
| 5.2 Were participants excluded due to missing data on intervention status? | There were no information about missing data | No information |
| 5.3 Were participants excluded due to missing data on other variables needed for the analysis? | There were no information about missing data | No information |
| 5.4 Are the proportion of participants and reasons for missing data similar across interventions? | There were no information about missing data | No information |
| 5.5 Is there evidence that results were robust to the presence of missing data? | Yes Outcome data is only 7. | Yes |
| Risk of bias judgement | No information about 2 participants and these could affect effect of starting and adhering to intervention. | Serious risk |

Domain 6: Risk of bias in measurement of outcomes

| Signaling questions | Comments | Response options |
| --- | --- | --- |
| 6.1 Could the outcome measure have been influenced by knowledge of the intervention received? | Knowledge of the intervention received could influence outcome measure. | Probably Yes |
| 6.2 Were outcome assessors aware of the intervention received by study participants? | Because of open label, there was no blinding | Yes |
| 6.3 Were the methods of outcome assessment comparable across intervention groups? | Outcome assessment is comparable across before and after. | Yes |
| 6.4 Were any systematic errors in measurement of the outcome related to intervention received? | There are not systematic errors in measurement of the outcome related to intervention received. | No |
| Risk of bias judgement | Outcome measurement were comparable but it could be influenced by knowledge of the intervention received by study participants and no information about blinding | Moderate risk |

Domain 7: Risk of Bias in selection of the reported result

| Signaling questions | Comments | Response options |
| --- | --- | --- |
| 7.1.Is the reported effect estimate likely to be selected, on the basis of the results from multiple outcome measurements within the outcome domain? | In Table 2, The PBQ is missing but author mentioned “There was a statistically significant improvement in bonding scores by measured PBQ. ( 20.00 -> 7.00, p= 0.010) | Probably No |
| 7.2 Is the reported effect estimate likely to be selected, on the basis of the results from multiple analyses of the intervention-outcome relationship? | Study did not report selectively and the pre-planning and the results are consistent. | No |
| 7.3 Is the reported effect estimate likely to be selected, on the basis of the results from different subgroups? | Study did not report selectively and the pre-planning and the results are consistent. | No |
| Risk of bias judgement | There is clear evidence that all reported results correspond to all intended outcomes, analyses. | Low risk |

Overall risk of bias

| Risk-of-bias judgement | Because Risk of bias due to missing data domain are serious risk  (at least one domain is serious -> serious risk) | Serious risk |
| --- | --- | --- |

Risk of bias assessment [Case study]

Tool for evaluating the methodological quality of case reports and case series

Safety and feasibility of repetitive transcranial magnetic stimulation (rTMS) as a treatment for major depression during pregnancy - Zhang, X. et al 2010

Domain 1: Selection

| Signaling questions | Comments | Response options |
| --- | --- | --- |
| 1.1 Does the patient(s) represent(s) the whole experience of the investigator or is the selection method unclear to the extent that other patients with similar presentation may not have been reported? | Patients could represent whole experience because she diagnosis the peripartum depression by DSM-IV. | Yes |

Domain 2: Ascertainment

| Signaling questions | Comments | Response options |
| --- | --- | --- |
| 2.1 Was the exposure adequately ascertained? | One session was applied per day and lasted two consecutive weeks. | Yes |
| 2.2 Was the outcome adequately ascertained? | HRSD score decreased from 35 to 12 | Yes |

Domain 3: Causality

| Signaling questions | Comments | Response options |
| --- | --- | --- |
| 3.1 Were other alternative causes that may explain the observation ruled out | She was antidepressant free and preferred receiving rTMS. | Yes |
| 3.2 Was there a challenge/rechallenge phenomenon? | After two months later participants was conducted second course | Yes |
| 3.3 Was there a dose–response effect? | No response because it is not drug event | * |
| 3.4 Was follow-up long enough for outcomes to occur | It is enough for outcome to occur | Yes |

Domain 4: Reporting

| Signaling questions | Comments | Response options |
| --- | --- | --- |
| 4.1 Is the case(s) described with sufficient details to allow other investigators to replicate the research or to allow practitioners make inferences related to their own practice | In this research, description was sufficiently detailed | Yes |

Overall risk of bias

| Risk-of-bias judgement | All domains are Low risk | Low risk |
| --- | --- | --- |

Safety and feasibility of repetitive transcranial magnetic stimulation (rTMS) as a treatment for major depression during pregnancy - Zhang, X. et al 2010

Domain 1: Selection

| Signaling questions | Comments | Response options |
| --- | --- | --- |
| 1.1 Does the patient(s) represent(s) the whole experience of the investigator or is the selection method unclear to the extent that other patients with similar presentation may not have been reported? | Patients could represent whole experience because she diagnosis the peripartum depression by DSM-IV. | Yes |

Domain 2: Ascertainment

| Signaling questions | Comments | Response options |
| --- | --- | --- |
| 2.1 Was the exposure adequately ascertained? | One session was applied per day and lasted two consecutive weeks. | Yes |
| 2.2 Was the outcome adequately ascertained? | HRSD score decreased from 35 to 12 | Yes |

Domain 3: Causality

| Signaling questions | Comments | Response options |
| --- | --- | --- |
| 3.1 Were other alternative causes that may explain the observation ruled out | She was antidepressant free and preferred receiving rTMS. | Yes |
| 3.2 Was there a challenge/rechallenge phenomenon? | After two months later participants was conducted second course | Yes |
| 3.3 Was there a dose–response effect? | No response because it is not drug event | * |
| 3.4 Was follow-up long enough for outcomes to occur | It is enough for outcome to occur | Yes |

Domain 4: Reporting

| Signaling questions | Comments | Response options |
| --- | --- | --- |
| 4.1 Is the case(s) described with sufficient details to allow other investigators to replicate the research or to allow practitioners make inferences related to their own practice | In this research, description was sufficiently detailed | Yes |

Overall risk of bias

| Risk-of-bias judgement | All domains are Low risk | Low risk |
| --- | --- | --- |

Antidepressant Effect of 58 Sessions of rTMS in a Pregnant Woman with Recurrent Major Depressive Disorder: A Case Report - Tan O et al, 2008

Domain 1: Selection

| Signaling questions | Comments | Response options |
| --- | --- | --- |
| 1.1 Does the patient(s) represent(s) the whole experience of the investigator or is the selection method unclear to the extent that other patients with similar presentation may not have been reported? | She could represent peripartum depression patients. | Yes |

Domain 2: Ascertainment

| Signaling questions | Comments | Response options |
| --- | --- | --- |
| 2.1 Was the exposure adequately ascertained? | The exposure adequately ascertained. (77 sessions) | Yes |
| 2.2 Was the outcome adequately ascertained? | HAMD-17 score was 31 to 4 | Yes |

Domain 3: Causality

| Signaling questions | Comments | Response options |
| --- | --- | --- |
| 3.1 Were other alternative causes that may explain the observation ruled out | Participant stop medication and start interventions | Yes |
| 3.2 Was there a challenge/rechallenge phenomenon? | No there was no rechallenge phenomenon | No |
| 3.3 Was there a dose–response effect? | No response because it is not drug event | * |
| 3.4 Was follow-up long enough for outcomes to occur | It is enough for outcome to occur | Yes |

Domain 4: Reporting

| Signaling questions | Comments | Response options |
| --- | --- | --- |
| 4.1 Is the case(s) described with sufficient details to allow other investigators to replicate the research or to allow practitioners make inferences related to their own practice | In this research, description was sufficiently detailed | Yes |

Overall risk of bias

| Risk-of-bias judgement | All domains are Low risk | Low risk |
| --- | --- | --- |

Repetitive transcranial magnetic stimulation for the treatment of major depression during pregnancy - Ferra˜o YA et al 2018

Domain 1: Selection

| Signaling questions | Comments | Response options |
| --- | --- | --- |
| 1.1 Does the patient(s) represent(s) the whole experience of the investigator or is the selection method unclear to the extent that other patients with similar presentation may not have been reported? | She could represent peripartum depression patients. | Yes |

Domain 2: Ascertainment

| Signaling questions | Comments | Response options |
| --- | --- | --- |
| 2.1 Was the exposure adequately ascertained? | The exposure adequately ascertained. (15 sessions) | Yes |
| 2.2 Was the outcome adequately ascertained? | The outcome adequately ascertained. (Use HDRS-21, HARS-14, CGI-S) | Yes |

Domain 3: Causality

| Signaling questions | Comments | Response options |
| --- | --- | --- |
| 3.1 Were other alternative causes that may explain the observation ruled out | Antidepressants could alternative interventions | No |
| 3.2 Was there a challenge/rechallenge phenomenon? | No there was no rechallenge phenomenon | No |
| 3.3 Was there a dose–response effect? | No response because it is not drug event | * |
| 3.4 Was follow-up long enough for outcomes to occur | It is enough for outcome to occur | Yes |

Domain 4: Reporting

| Signaling questions | Comments | Response options |
| --- | --- | --- |
| 4.1 Is the case(s) described with sufficient details to allow other investigators to replicate the research or to allow practitioners make inferences related to their own practice | In this research, description was sufficiently detailed | Yes |

Overall risk of bias

| Risk-of-bias judgement | Causality domain is unclear risk | Unclear risk |
| --- | --- | --- |

Use of repetitive transcranial magnetic stimulation for the management of bipolar disorder during the postpartum period - Cohen RB et al 2008

Domain 1: Selection

| Signaling questions | Comments | Response options |
| --- | --- | --- |
| 1.1 Does the patient(s) represent(s) the whole experience of the investigator or is the selection method unclear to the extent that other patients with similar presentation may not have been reported? | She could represent peripartum depression patients. | Yes |

Domain 2: Ascertainment

| Signaling questions | Comments | Response options |
| --- | --- | --- |
| 2.1 Was the exposure adequately ascertained? | The exposure was not adequately ascertained. (10 sessions) | Yes |
| 2.2 Was the outcome adequately ascertained? | The outcome adequately ascertained. (Use HDRS-21, YMRS) | Yes |

Domain 3: Causality

| Signaling questions | Comments | Response options |
| --- | --- | --- |
| 3.1 Were other alternative causes that may explain the observation ruled out | Other alternative causes were ruled out | Yes |
| 3.2 Was there a challenge/rechallenge phenomenon? | Patients returned with symptoms of depression after 3 days So rechallenge 800 pulses on the left and 800 pulses on the right DLPFC) | Yes |
| 3.3 Was there a dose–response effect? | No response because it is not drug event | * |
| 3.4 Was follow-up long enough for outcomes to occur | It is enough for outcome to occur | Yes |

Domain 4: Reporting

| Signaling questions | Comments | Response options |
| --- | --- | --- |
| 4.1 Is the case(s) described with sufficient details to allow other investigators to replicate the research or to allow practitioners make inferences related to their own practice | In this research, description was sufficiently detailed | Yes |

Overall risk of bias

| Risk-of-bias judgement | Causality domain is unclear risk | Unclear risk |
| --- | --- | --- |

Repetitive transcranial magnetic stimulation (rTMS) in major depressive episode during pregnancy - Klírová M et al 2008

Domain 1: Selection

| Signaling questions | Comments | Response options |
| --- | --- | --- |
| 1.1 Does the patient(s) represent(s) the whole experience of the investigator or is the selection method unclear to the extent that other patients with similar presentation may not have been reported? | She could represent peripartum depression patients. | Yes |

Domain 2: Ascertainment

| Signaling questions | Comments | Response options |
| --- | --- | --- |
| 2.1 Was the exposure adequately ascertained? | The exposure was not adequately ascertained. (15 sessions) | Yes |
| 2.2 Was the outcome adequately ascertained? | The outcome adequately ascertained. (Use MADR’s score, Beck score) | Yes |

Domain 3: Causality

| Signaling questions | Comments | Response options |
| --- | --- | --- |
| 3.1 Were other alternative causes that may explain the observation ruled out | Antidepressants could apply as co-intervention | No |
| 3.2 Was there a challenge/rechallenge phenomenon? | There were no challenge/rechallenge phenomenon | No |
| 3.3 Was there a dose–response effect? | No response because it is not drug event | * |
| 3.4 Was follow-up long enough for outcomes to occur | It is enough for outcome to occur | Yes |

Domain 4: Reporting

| Signaling questions | Comments | Response options |
| --- | --- | --- |
| 4.1 Is the case(s) described with sufficient details to allow other investigators to replicate the research or to allow practitioners make inferences related to their own practice | In this research, description was sufficiently detailed | Yes |

Overall risk of bias

| Risk-of-bias judgement | Causality domain is unclear risk | Unclear risk |
| --- | --- | --- |
